# Methodology to Improve Design of Accelerated Life Tests in Civil Engineering Projects

**DOI:** 10.1371/journal.pone.0103937

**Published:** 2014-08-11

**Authors:** Jing Lin, Yongbo Yuan, Jilai Zhou, Jie Gao

**Affiliations:** Department of Construction Management, Dalian University of Technology, Dalian, China; Coastal Carolina University, United States of America

## Abstract

For reliability testing an Energy Expansion Tree (EET) and a companion Energy Function Model (EFM) are proposed and described in this paper. Different from conventional approaches, the EET provides a more comprehensive and objective way to systematically identify external energy factors affecting reliability. The EFM introduces energy loss into a traditional Function Model to identify internal energy sources affecting reliability. The combination creates a sound way to enumerate the energies to which a system may be exposed during its lifetime. We input these energies into planning an accelerated life test, a Multi Environment Over Stress Test. The test objective is to discover weak links and interactions among the system and the energies to which it is exposed, and design them out. As an example, the methods are applied to the pipe in subsea pipeline. However, they can be widely used in other civil engineering industries as well. The proposed method is compared with current methods.

## Introduction

### 1 Reliability Design

#### 1.1 Reliability vs. Quality

Quality and Reliability are two major concerns in the engineering industry, especially for manufacturing and civil engineering. Quality relates to a product or structure's initial performance when new, while reliability relates to performance over its lifetime. Over the years many industrial standards and evaluation systems have been built relating to quality, such as Six Sigma, ISO 9000, the Malcolm Baldrige National Quality Award and the Deming Prize. The American Society for Quality (ASQ) has garnered well over 150,000 members and continues to grow in strength. By comparison reliability is fairly neglected; the Institute for Reliability Engineering languishes is near obscurity. However, for the end customer reliability is equally, if not more important than quality because of future maintenance costs and the consequences of potential failures in today's large, complex systems.

In this paper we focus on reliability. We propose new and more systematic methodologies to discover destructive energies and apply the methods to the pipe in a subsea pipeline for demonstration purposes. In addition to incorporating prior experience in a design to avoid known failure mechanisms, reliability can be improved by exploring for unknown weak links and interactions resulting from the multiple, simultaneous stresses that a pipeline may experience during its operational life. The pipe should survive the compound impact from both external environment and internal stresses throughout its designed lifetime, say, for 20 years or more.

#### 1.2 Monumental economic loss of due to lack reliability

Keki Bhote in his book, *World Class Reliability* states, “Many companies take a cavalier attitude in dismissing their costs associated with reliability. They express their satisfaction if warranty costs are within a few percent of their sales dollars. They may justify such costs by including them in their budgets. Tragically, most companies do not recognize that their own warranty cost is only the tip of the iceberg” [1]. The total cost of reliability failures includes not only direct repair costs but also the costs of supplier retrofits, lawsuits, and third party costs to their customer's customers, to society, to governments and environmental damage. These costs are virtually never included in an economic analysis.

James R. Chiles in his book, *Inviting Disaster; Lessons from the Edge of Technology*, cites numerous examples where simply design changes could have avoided many high visibility catastrophic failures [2]. To wit, the 1979 partial meltdown of a Three Mile Island nuclear power plant reactor, the 1980 capsizing of the *Alexander Keilland* floating platform to house offshore oil workers, the 1982 sinking of the *Ocean Ranger* offshore floating drill rig, the 1986 explosion of the *Chernobyl* nuclear power station, the 1986 mid-air breakup of the Space Shuttle *Challenger*, the 1988 explosion and fire on the *Piper Alpha* offshore drilling rig, the 1999 loss of the *Mars Polar Lander*, the 2000 crash of the *Air France Concord* departing Charles de Gaulle Airport, and many others. Since the publication of his book, the litany of such disasters continues with such high profile events as the 2010 *Deepwater Horizon* oil spill in the Gulf of Mexico and the 2011 meltdown of the Fukushima nuclear power plant following an earthquake. Many of these disasters could have been avoided with adequate stress testing to find the weak links and designing them out, or in some cases, simply not ignoring known weak links.

### 2 Weaknesses of current practices regarding reliability

#### 2.1 Logic-based approaches for risk factor identification

Many logic-based approaches have been applied to reliability risk analysis. Fault Tree Analysis, FTA, has been used for identifying and ranking risk factors based on their assigned probabilities [3]. Bayesian Belief Networks build a network used to demonstrate the logic relationship between factors through a probability distribution calculation [4]. Failure Mode and Effects Analysis, FMEA, is used to evaluate the effect of potential failures of designed functions [5]. Cause and defect Diagrams, also called Fishbone Diagrams, are used to identify potential factors which would cause the system to fail [6], etc. All these methods are based on knowledge and experience; factors are often identified through brainstorming. They are useful early in the design phase to prevent incorporating failure modes which have previously been discovered or which are suspected. However, their weakness lies in the fact that unknown factors and interactions are missed – the very factors that are often responsible for so-called “rare events” or “Black Swans” [7].

#### 2.2 Mathematical modeling

Reliability testing often includes mathematical modeling. These models are typically expressed graphically or with mathematical functions. Researchers design experiments to simulate the relationship between the input parameters/factors and the major output functions. In order to increase fidelity of the model, complexity is incrementally added following analysis and inference, or from other data. But it is impossible to predict with certainty whether a given mathematical model is adequate or not [8].

Other fundamental weaknesses of the mathematical approach are: 1) Large sample sizes required to test the model. For example, based on the binomial proportion confidence interval, doubling the number of failure modes will triple the sample size [9]. 2) Much time is consumed for direct testing to a significant portion of life. 3) High confidence levels are difficult to achieve (50% is meaningless, 70% is not a whole lot better, 90–95% is desirable) because the quantities, costs and test times become astronomical as confidence level requirements increase.

Having noted the deficiencies of mathematical modeling, we hasten to point out that such models are invaluable for functional design *before* life testing begins. In this situation designers are trying to select and apply the physical laws of nature necessary to make the system function as intended. However, in the case of reliability it is more important for designers to prevent *unexpected* behavior of the system. This is a much broader challenge and requires a different experimental strategy, such as accelerated life testing.

#### 2.3 Accelerated lifetime tests

Accelerated life testing (ALT) attempts to bring out unexpected weak links and interactions in a design. These are failure modes that were not or could not be predicted by prior modeling, design and prototyping. Various ALT methods are in use, such as HALT (Highly Accelerated Life Testing) and HASS (High Accelerated Stress Screen) [10] which apply two basic stresses, temperature and vibration typically, in parallel or sequentially, along with other special stresses associated with the system's operation. Another example is HAET (High Accelerated Environmental Testing), in which a stimulation mechanism is introduced in order to quantify the factors/failure modes at “end of life”.

However, there are certain shortcomings of most ALT methods, which may include: 1) Environmental stresses are often assumed to be single-factor effects, not combined and not interacting. 2) Over-stress levels could be insufficient to detect the most important failure modes. 3) Incremental stress step size could be too small to yield results within a suitable time period or too large and skip over important failure modes. 4) Excessive stresses may introduce artificial failures which would never occur in the field. 5) A large number of test units are needed for each failure mode. 6) Converting time-to-failure to a calculated lifetime is risky because overstressing is likely to behave in an unpredictable, non-linear way, depending on the overstress levels applied.

Our preferred approach is to use Multiple Environment Over Stress Testing (MEOST) primarily because it avoids shortcomings 1–5, and does not attempt to predict lifetime as #6 above. Rather it focuses on assuring that full life can be reached at full stress without experiencing unexpected failure modes from weak links and interactions. The method is briefly described below, and we develop new methods to assure more systematic discovery of the energies that should be fed into an MEOST experiment.

#### 2.4 Function Models in reliability

Function models are often used to explain in graphical form how a system is supposed to work in terms of how its subsystems link and operate together to achieve the overall goal(s). Commonly used function models are: Function Tree, which builds the function structure by decomposing the major function(s) into individual sub-functions based on the logic relationship between them [11]; Function Analysis System Technique (FAST), a similar method introduced by the Society of American Value Engineer in 1965 [12]; Structured Analysis and Design Technique (SADT) employs a diagrammatic notation designed specifically to help people describe and understand systems [13].

Such models are excellent from the perspective of developing the required system functionality and, given good design, components and assembly, the initial system quality should be good. However, from a reliability perspective there is an important aspect which needs to be added: energy or energy combinations which cause failure. In particular we are concerned with (a) external/environmental energies, (b) energies employed in the system which lead to stresses on the system, and (c) energy losses which occur as energy is transferred among system sub-functions. The second law of thermodynamics assures us that some energy will be lost during transformation; part of that lost energy may go into destructive behavior.

## Methods

### 1 Energy-Strength-Decay Model

**Figure 1:**
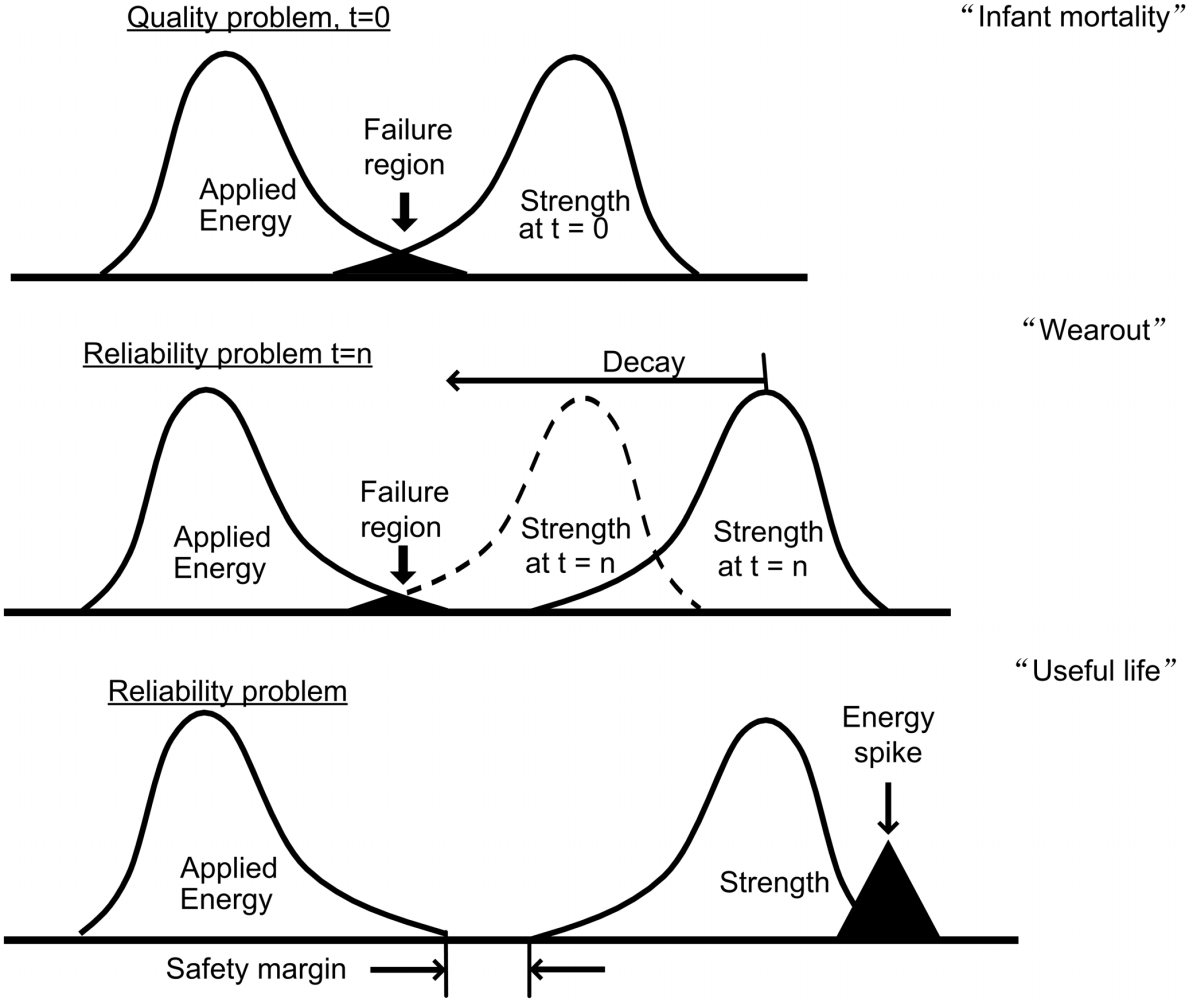
Energy-Strength-Decay Model. A conceptual model of a system's strength to resist energy sources. Due to natural variability, each is shown as a distribution. As long as there is a safety margin between them, the system will function without failure. Three types of failures are shown in the graphic. See text for a description of each.

In order to visualize the relationship between energy-to-destroy and strength-to resist we employ the Energy, Strength and Decay (ESD) model shown in Fig. 1. “Energy” can be any force or energy source, such as pressure, temperature, weight, etc. In the figure both energy and strength are shown as distributions because of natural variability. Reliability failures occur in a number of different ways, and it is useful to segment these into three categories, “Infant mortality” and “Useful life”, and “Wear out” [14].

When a system is first built (t = 0), we expect the energy and strength distributions to be completely separated with a reasonable safety margin in between. If the distributions overlap as shown in the top diagram of Fig. 1, the strongest energies will break the weakest systems, even when the system is new. This is a quality problem because initial strength did not meet the energy design criteria. We say such systems failed as infants hence the term “Infant Mortality”.

In the middle diagram there is no overlap at t = 0. The quality is good; initial strength is able to withstand all the energies with a safety margin. However, over time the strength distribution decays as system components wear out. The strength distribution moves to the left until it overlaps the energy distribution and the weakest systems (or those which have decayed the most) fail. This is a “Wear Out” reliability failure.

The bottom diagram in Fig. 1 shows the energy and strength relationship during the system's “Useful Life”. Energy and strength distributions do not overlap so failures should not occur. But occasionally they do. This type of failure is characterized by an excessive energy spike which totally engulfs the strength distribution. For example, a car crashes into a tree and is destroyed by its forward momentum. Obviously, if one could make the car infinitely strong it would not fail during the crash. However, this is neither economic nor practical. Other strategies are needed to deal with this situation. For now, the best we can do is design in a suitable safety margin and test up to and somewhat beyond that margin using multiple energies to discover any weak links which might impact quality or reliability.

From the Energy-Strength-Decay model, we see that quality failures may occur at any time during life. It may be that initially weak systems were never exposed to maximum environmental and internal operating energies, but later in life they are, and failure occurs at that time. Careful inspection of the failure mode will determine whether strength did not meet the initial requirement (a quality problem), whether failure was due to an excessive energy spike (useful life) or due to decay of system strength (a wear out reliability problem). Clearly distinguishing these situations is critical to developing proper corrective actions.

### 2 MEOST

#### 2.1 MEOST Methodology

The MEOST method of accelerated life testing simply applies multiple energies in increasing stepwise fashion to reveal weak links in a system's strength related to Infant Mortality or Wear Out (but not to Useful Life excessive energy spikes). Once these weak links are discovered they can be designed out by investigating their root cause and strengthening the system against that combination of energies. In one of the first applications of MEOST a subcontractor of NASA applied it to the *Apollo* Lunar Excursion Module, the LEM [15]. The specification included operability up to 95% relative humidity. However, over stress tests were run up to 100% humidity, a dripping wet environment, and the electronics were improved to withstand this stress. This safety margin prevented electrical failure in the LEM during *Apollo 13* when the crew had to retreat there during the return trip from the moon; cold temperature caused moisture from the astronaut's breath to condense on the electronics, an unexpected stress. These days, MEOST is becoming popular, especially in the electronics and automotive industries. There are even companies which specialize in running MEOST as a service, such as Intertek.com.

**Figure 2:**
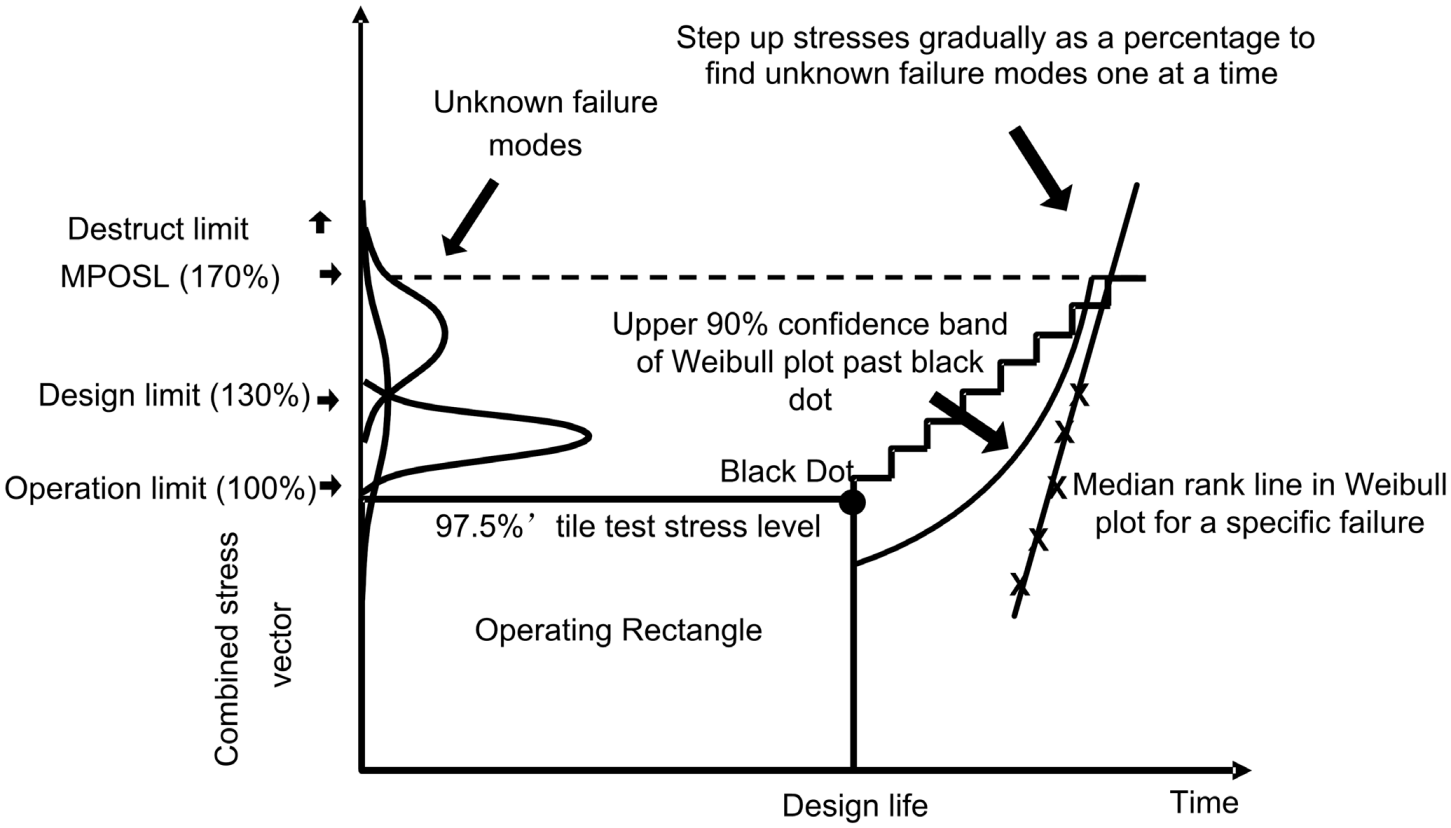
MEOST Operating plan graphic. Testing first proceeds inside the “Black Dot Operating Rectangle” which is defined as 97.5% maximum combined stresses over the design life, and then beyond the rectangle by overstressing in time-steps. Individual failure modes are identified by root cause analysis and plotted on a Weibull plot to determine if their 90% confidence limit will intersect the Operating Rectangle before the design life is reached at max stress.

Briefly as seen in Fig. 2, the MEOST plan is to operate the system within its operating limits for a period of time while exposing it to all energy types simultaneously. Time should be compressed between cycles so that the system experiences the largest possible fraction of stress cycles expected over its design life. For example, if oil flow in a pipeline is expected to change once a week for 20 years, this is about 1,000 cycles. By cycling flow once every 15 minutes the entire life relative to this stress can be simulated in a little over 10 days. This strategy is time acceleration. Flow rate would go from max to standstill and back, but at normal rates of change (no energy spikes). The “Black Dot” represents 97.5% of the combined maximum stresses (operating plus environmental) applied throughout the design life.

However, time acceleration may not be able to cycle all the stresses expected to occur over the system life. So after a period of time we move beyond the Black Dot to overstressing, trying to bring out remaining weak links. Using the same cycling profiles all stresses are applied at proportionally higher levels and proportionally faster rates of change. Overstressing is applied in small incremental steps so as not to skip over failure modes. Whenever a part fails, the failure mode is carefully inspected to determine root cause, and the time-to-failure is recorded. After multiple parts have failed with the same failure mode, these are plotted on a Weibull plot and the 90% confidence limit is determined for that failure mode [16]. If the 90% confidence limit intersects the Operating Rectangle before the full Design Life at 97.5% combined stresses, the failure mode has to be fixed. If not, the system is safe at the 90% confidence level against that failure mode over the course of its designed stress and life.

MEOST testing is typically 1–2 orders of magnitude faster than normal stressing in bringing out latent failure modes. However during overstressing, failures may be 2–3 times worse than normal and occasionally they may be catastrophic. If possible, energy limitations should be included to avoid destroying all the evidence of the initiating feature so that root cause can be determined and system strength improved accordingly.

#### 2.2 Planning an MEOST Experiment

Like all experimental methodologies, good preparation is essential for the best results. Energies and life time goals should be specified. Minor failures should be eliminated before testing – there is no point in using a powerful method to discover simple things that should have been avoided beforehand. Stress levels and cyclical profiles should be identified for normal operating limits. There are also the design limits, the Maximum Practical Over Stress Levels (MPOSL) [17], and beyond that the destruct stresses. Design limits include the safety margin beyond the maximum operating limits. MPOSL could be taken, for example, as 170% of the operating limits, but less than the destruct limits. Also needed are the step sizes for each overstress, the dwell time at each stress step and the time sequencing of stresses to simulate real life. Finally, a test setup capable of applying simultaneous stresses up to the MPOSLs should be designed and built, and it should be able to handle the required test sample size (typically 10–20 parts).

A typical sequence of test steps is described by Bhote [17]. We have modified these for civil engineering applications as follows:

Step 1: Apply each energy separately to its design limit (operating limit plus safety margin). Fix any failures.

Step 2: Apply the combined energies up to 97.5% of the operating limits (Black Dot stresses) in the way these are expected to occur during life, but accelerated in time as much as possible without violating anticipated maximum rates of change.

Step 3: Apply the combined stresses in 2–4 steps increasing from the operating limits up to the design limits.

Step 4: Apply each stress separately to its MPOSL.

Step 5: Apply the combined stresses in 4–5 steps increasing from the design limits to the MPOSL limits.

During initial testing one may find more than one failure mode among the failed samples. Testing may continue without fixing these failure modes up until a specific failure mode is found to dominate the test sample population. Then all failure modes discovered up to that point should be eliminated and testing restarted from the beginning with improved samples to find the next weakest link(s). Testing stops when the Weibull plot for the weakest uncorrected failure mode does not intersect the Operating Rectangle with 90% confidence out to the design life (Black Dot in Fig. 2).

Follow-up testing may continue after field installation, if practical, by bringing back parts that have been in use for 6 months or more and subjecting them to MEOST. The reason for recommending this step is that a failure mode could be discovered due to some operating or environmental condition which was not included in the original MEOST plan. For example, when the company Hamilton Standard switched from manufacturing wood propeller blades for airplanes to making them of aluminum, some blades were brought back from selected customers in the field after six months of use. In exchange, those customers received new blades for free. The returned blades were tested and found to be corroded from the inside, something not visible from the outside. It was an undiscovered wear out failure mode. Competitors who did not do such field buy-backs subsequently had propeller blades fail in flight, and went out of business due to lawsuits. Reliability testing, including buy-backs, was a lot cheaper in the long run.

Chiles in his book *Inviting Disaster* says, “I like to think of thorough testing as a badge of confidence: confidence that the machine can take abuse, and if it breaks, the designers will be able to find a solution. It is good business, reassuring users that there is no place they can go that the manufacturer hasn't already been.” [18]

## Proposed New Methods

Failure simply means the system was not strong enough to withstand the energies it was exposed to over time. Clearly, then, before strengths can be designed the energies to be endured must be identified. We propose an innovative two step approach to find all the energies: an Energy Expansion Tree (EET), to systematically discover external/environmental energies, and an Energy Function Model (EFM) to discover internal or system energies. All these energies should be included in a MEOST experiment to simulate real life. Hidden and unknown weak links, once found experimentally, need reparation if they adversely intersect the Operating Rectangle.

### 1 Energy Expansion Tree

The conventional approach to finding external energies would be to brainstorm a list of them. However, we look for a more systematic approach. To be more objective and complete we construct an “Expansion Tree” by following a set of principles based on physical, geometrical and logical relationships in such a way that it is unlikely to overlook any energy. Previously, we have applied a similar method to construct Fault Trees and this is described in more detail elsewhere [19].

#### 1.1 Expansion Tree Design principles

The design principles for Expansion Trees, modified for application to discovering energies and forces, are as follows:

Principle #1: No bias/prejudgment. Expert knowledge leads one to simply list known or suspected energies. Reliability is about trying to discover what is unknown, so a more systematic approach is needed.

Principle #2: Nodes at each level should be mutually exclusive, in other words, independent of each other. Overlapping content obscures judgment of completeness.

Principle #3: Nodes at each level should be collectively exhaustive, in other words they should add up to 100% of the possibilities from the node immediately above.

Principle #4: To make principles #2 and 3 more rigorous a “basis” of split should be stated at each level against which to judge independence and completeness.

Principle #5: Decompose each node until an energy is reached which can be included in the MEOST experiment, such as temperature, pH or a pressure.

Principle #6: Split levels should be as symmetric as possible to provide level independence. This rule should be regarded as a guideline to organizing the vertical development of a tree.

These six principles assure maximum node coverage with a low probability of leakage (i.e., missed energies). Following them regulates the thought process on how to group things in a more consistent and logical way.

#### 1.2 Energy Expansion Tree for a subsea pipeline system

**Figure 3:**
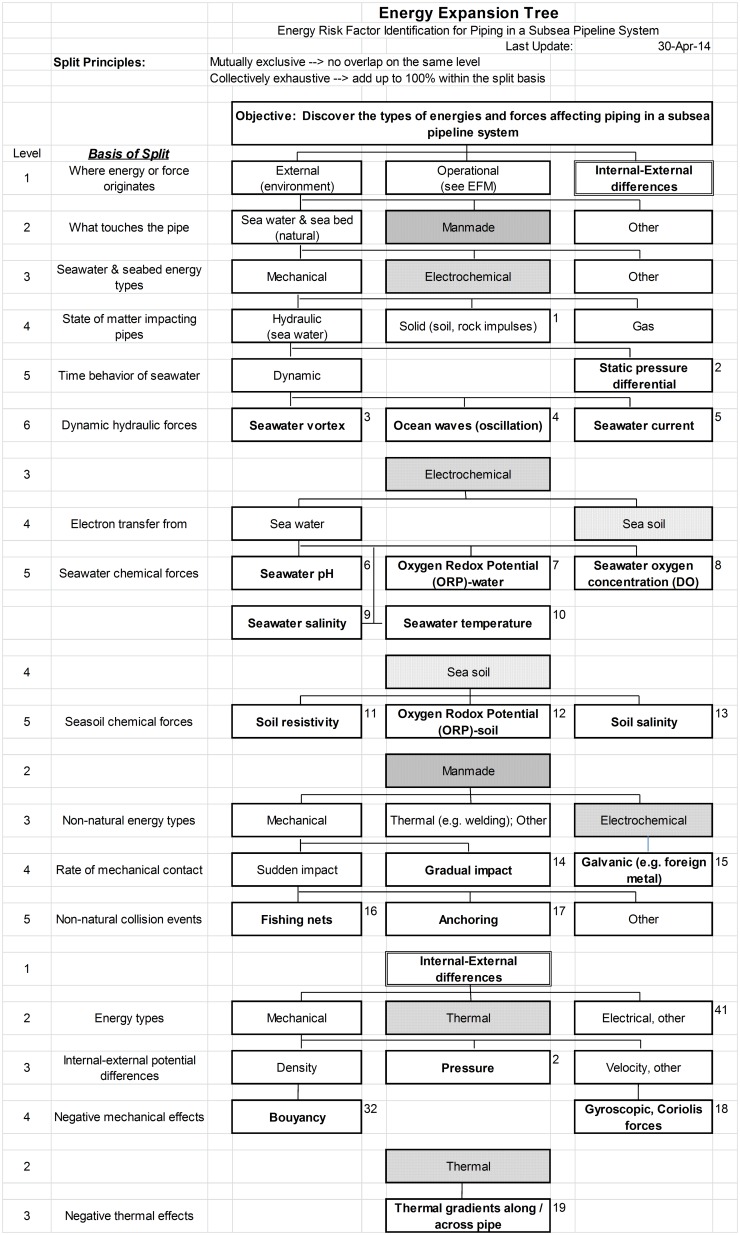
Energy Expansion Tree as applied to piping in a subsea pipeline. Boxes with bold font represent the energies to be fed into MEOST. Boxes with various shaded backgrounds and borders are used to distinguish the corresponding branch development underneath it. Numbers to the right of bolded boxes are used for linking the energies into the subsequent MEOST table.

Following the above principles, we develop the Expansion Tree for energies related to the piping in a subsea pipeline as shown in Fig. 3. The nomenclature, “Energy Expansion Tree” (EET) is chosen because this logic tree is concerned solely with energy, regardless of the reliability regime. Three general categories are identified which cover all the energy sources related to a subsea pipeline: (a) “External energy sources” outside the system (the environment), (b) “Operational energy” for energies employed in and used by the system itself, and (c) “Internal-External Differences” resulting from the 2nd law of thermodynamics which gives rise to equilibration forces whenever two regions of space have different energy levels. The external and internal-external difference branches are split into sublevels using the “basis of split” shown in the leftmost column according to expansion tree principles, while the internal (operational) branch is derived below from a newly proposed energy function model. The reason different approaches are needed is that external to the system the environment is unstructured, while the system itself is designed with very specific laws of physics in mind in order to achieve the system primary functions. The later can be enumerated by design; the former cannot.

In this example the tree is applied specifically to a subsea pipeline system to be built in the China South Sea. Environmental energies are therefore limited to tropical water conditions. Thus for example, “floating ice” is not considered, but would be a significant potential energy source if the pipeline were to be built in the North Sea. Below are some examples of how the decomposition proceeds.

Mechanical impact from things that touch the pipe could be in the form of solids, liquids (hydraulic) or gas. Solid impact would come from soil/rock shifting and would occur as impulse forces, although these may not retrace to back to zero. Hydraulic impact could be static or dynamic, and following the “collectively exhaustive” rule, dynamic hydraulic forces would arise from “Seawater vortex”, “Ocean waves (oscillation)” and “Seawater current”. Note that traditionally one might think to describe hydraulic energies in terms of their causes, such as “Tsunami” or “Hurricane”, but these are energy sources, not energy types. The initial objective is only to think of energy types. Later, when developing energy amplitudes and profiles, one would consider sources.

Under “Electrochemical”, we look for things that touch the pipeline because electrochemical energies come from electron exchange. There are only two possibilities, “Sea water” and “Sea soil”. Most research indicates that five factors determine the electrochemical condition of ocean water: “pH”, “Oxygen Redox Potential (ORP)”, “Oxygen concentration”, “Salinity” and “Temperature”. These factors are interrelated and X Tang and J Wang provide a detailed analysis of their interactions [20]. pH is primarily driven by H_2_CO_3_ in the sea water [21]. Following Palmer et al [22], under “Sea soil” we include “Soil resistivity”, “Oxygen Redox Potential (ORP)” and “Soil salinity”.

Splitting “Manmade” is potentially more challenging because there are many ways humans could introduce stresses to the pipeline, whether accidentally or on purpose. We begin by thinking about general types of energies. Under “Mechanical” a logical split based on speed of impact is made. Sudden “bumping” up to the design limit could occur, such as “Fishing nets”, “Anchoring” and other ocean activity. “Gradual impact” might include a snagged fishing net whose force would behave asymptotically if a net got stuck while being pulled up. In any case, these examples suggest both sudden and gradual mechanical forces should be fed into the MEOST experiment, with amplitudes to be determined more by design specs than limitless overstresses. Sensible Maximum Practical Over Stress Limits (MPOSL) should be selected above the design criteria. Destruct limits beyond that would result from failure modes which are intentionally excluded, such as solder melting on a printed circuit board. These are more appropriately considered under Useful Life failures.

The third general category, “External-internal differences” is normally prepared after completion of the Energy Function Model (see below). This is an example of an entire set of forces which might be overlooked without constructing an EET following the design principles and thinking about the basic laws of nature.

In this branch, density, pressure and velocity are identified as mechanical differences between internal and external regions of space (inside vs. outside the pipe). Oil density vs. seawater gives rise to buoyancy forces. Velocity differences from oil movement result in gyroscopic and Coriolis forces. While these two forces are small compared to other mechanical forces, here it is important that we identify and consider all possible forces. It is one thing to objectively ignore forces because they are small, but something altogether different to miss forces entirely. Finally, thermal differences give rise to thermal gradients as the oil in the pipeline is heated vs. external seawater.

Enumerating energy types is the first step in developing an effective MEOST experiment. To our knowledge, this is the first time in the oil pipeline industry that a systematic approach has been applied to discovering environmental energies, with the intention of looking for weak reliability links in systems under multiple stress conditions. Next we look for energies and forces that arise with the system itself.

#### 1.3 Energy Function Model

To understand energy sources in the “Operational energy” branch, we begin by constructing a standard Function Flow Model (FFM) for the subsea pipeline system. The objective of this model is to understand how the system works. Like all function models, the horizontal direction shows HOW a function is achieved (reading left to right), or inversely WHY the function is needed (right to left). The vertical or WHEN direction shows the consequences of functions, which often lead to additional design considerations or even new subsystems. Horizontal logic is either “or” or “and”, and control knobs are added to manage both functionality and its consequences.

**Figure 4:**
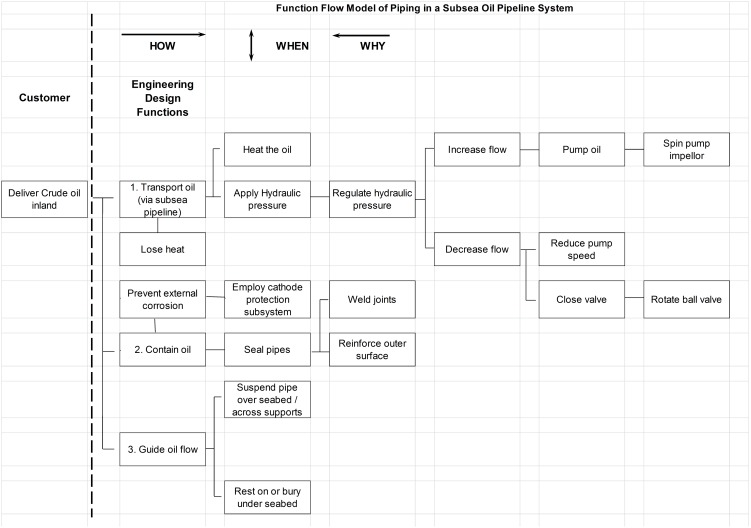
High-level Function Flow Model (FFM) for a subsea pipeline system. The function model shows three primary functions required to deliver oil inland. Each box states a function (a verb) and boxes to its right explain HOW that function is achieved. Vertical linkages (the WHEN direction) show consequences of functions.

In the high level Function Model for a subsea pipeline seen in Fig. 4, there are three primary functions: “Transport Oil”, “Contain oil” and “Guide oil flow” (a path). These decompose from left to right. Each function (the verb in the box) is driven by the function immediately to its right, until the lowest order function is reached.

From this model we see that energies in the system are primarily stored and used in the form of pressure, heat and velocity (kinetic energy and momentum). Under Transport oil the system operates by applying pressure to push the oil through the pipeline and it applies heat to make the oil less viscous and easier to push. Under “Contain oil” we see that pipes are used. Pipes will corrode and a cathode protection subsystem is employed to mitigate that. Pipes also have weight, which doesn't matter from a functional perspective, but later, from an energy perspective it does because weight contributes to mechanical stresses. These are clearly linked to the pipe support structure seen under “Guide oil flow”. Under normal circumstances these stresses are static, however if environmental conditions change they may become dynamic, at least momentarily.

**Figure 5:**
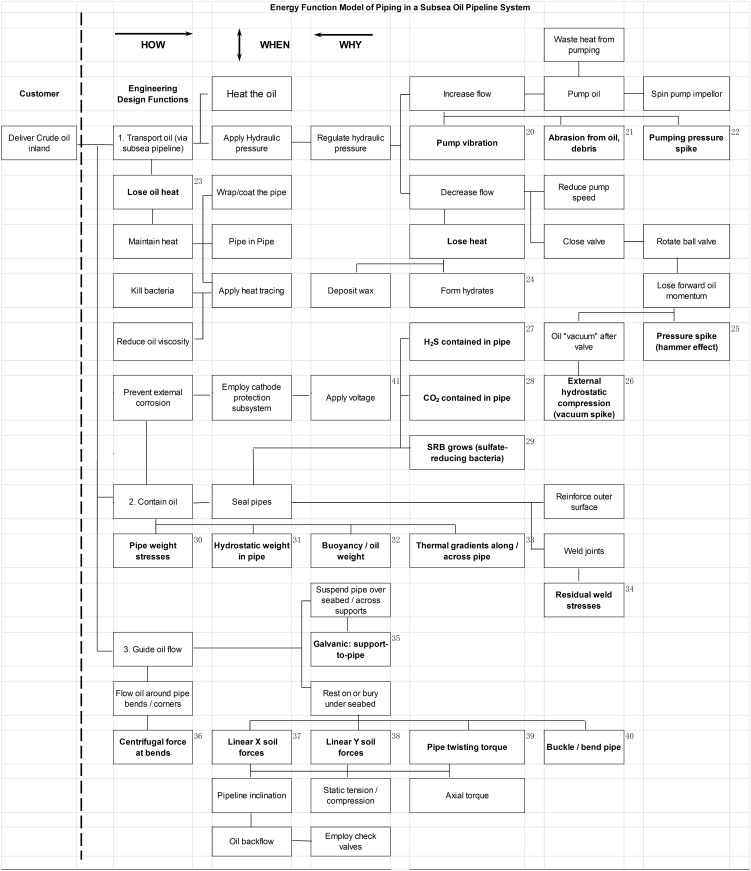
Energy Function Model (EFM) for piping in a subsea pipeline system. The Function Flow Model of Fig. 4 expanded to include system energies. Cells with Bold font are direct energy side effects associated with the designed functions or other cells immediate above/below. Bolded cells are fed forward into the MEOST experiment using the energy codes to the right of each bolded box.

The next step is to identify energy losses and other forces which result from the existence of the pipeline. These can be regarded as side-effects, so we add them in the WHEN direction. They are important from a reliability perspective because they produce stresses and wear. Thus we build the integrated Energy Function Model (EFM) shown in Fig. 5.

In the figure, we see that while transporting oil in a pipe, heat gets lost. In the initial design this led to taking corrective action to maintain heat (a secondary function), such as adding electrical heating and wrapping the pipe to insulate it. Transporting oil requires pressure to move it and when moving, oil has considerable momentum (mass times velocity). “Increase flow” and “Decrease flow” cover these aspects of pumping and momentum changes. Specifically, three negative mechanical energies from increasing flow are, “Pumping vibration”, “Abrasion from oil, debris” and “Pumping pressure spike”. Pump vibration energy is transmitted either through the pipe itself or the oil inside. Under “Decrease flow” a valve closing too quickly (or any sudden blockage) would create a pressure spike (hammer effect) on one side of the valve or blockage and a pressure drop (vacuum spike) on the other side. These forces may be enormous due to the large moving mass in the first case and the external water pressure at depth in the second case. “Decrease flow” creates other side effects as well, namely “Lose heat”, which leads to the well-known problems of “Deposit wax” and “Form hydrates” [23].

In a similar way, the functions “Contain oil” and “Guide oil flow” are developed to assist in defining what energies should be applied in the simulated testing. For example under “Guide oil flow”, “Centrifugal forces” as oil flows around bends result in tension to the adjoining straight pipe sections and sideway forces on the pipe wall at the bends. Contact with the soil may result in X-Y linear forces, twisting torques or buckling forces as the soil shifts over time.

The Energy Function Model completes the second major branch, “Operational energy”, of the EET in Fig. 3, and thus the energy identification stage. To summarize, the EET and EFM are developed only to discover the types of energies and forces that should be applied in a MEOST fixture. They do not describe time-lines and operating profiles, which are also necessary for actual simulation. Nor are they meant to suggest corrective actions or remediation to compensate for negative energies in the design, although if found, corrective actions are certainly warranted, possibly in terms of adding new subsystems.

Note that each subsystem, such as the cathode protection (CP) subsystem or the support structure subsystem, has its own set of primary functions to be protected, energies-to-destroy and strengths-to-resist. These must be evaluated and treated with separate MEOST experiments to find their weak link failure modes. Where subsystems interact, energies should be included in the subsystem being studied. In the current example the CP subsystem creates electric fields and the supports create galvanic forces.

The list of energies from a systematic discovery process such as we have employed may be rather extensive. But an MEOST experiment cannot be done with 100 separate energies. Typical they only include dozen or so. Therefore the next step is to consolidate energies in such a way that their overall effect still represents real life as much as possible.

## Results and Discussion

### 1 Application of MEOST experiment for Subsea pipeline reliability design

In the subsea pipeline project of the National Program 973 [24], the pipe samples used are low carbon stainless steel with the following parameters: Outer Diameter D = 0.354 m, Wall thickness t_P_ = 1.2 cm, Material density ρ_P_ = 7800 kg/m3, Young Modulus E_P_ = 2.07 GPa, Poisson ratio μ = 0.28, Yield stress σs = 387.9 MPa, Length = 6 m sections, Sections welded together for construction simulation.

Historically, the reliability test plan for an undersea pipeline focuses on corrosion because experience has shown this is a high reliability risk. The Program 973 team set up a test stand to measure the pipe stress-strain curve (tensile curve) under different corrosion conditions. The Poisson ratio (μ), Young Modulus (E) and Yield stress (σs) can be calculated and evaluated based on various algorithms. Through Finite Element Analysis and other software, a physical model is developed to describe the relationship between pipe strength and corrosion. Great effort and cost have been invested to simulate corrosion effects because of the potential cost consequences should the pipe leak or break. However, the focus on pipe corrosion itself should not lead to “tunnel vision” in which other potential failure modes are ignored, either of the pipe itself, or for that matter of other pipeline subsystems such as the cathode protection and pipeline support subsystems. Interactions among subsystems could lead to chain-of-event type failures. One of the reasons MEOST is proposed here is that it does not focus on any particular failure mode of the subsystem being studied. It merely tries to find the next weakest one during design life. If that one intersects the Operating Rectangle then remedial strengthening action has to be taken to avoid a disaster, just as much as preventing or reducing corrosion.

### 2 Design of the subsea pipeline MEOST experiment

The example MEOST experiment we develop here is for the piping subsystem of a subsea pipeline discussed above. It would employ 10–12 pipe samples tested simultaneously, but scaled down to 10–20% of full size. Unlike the corrosion test fixture, the MEOST test fixture would include support structures, cathode protection and other devices which, together, produce the energy combinations found earlier in the pipe subsystem EET and EFM.

Forty one different energy types were identified which could affect pipe reliability during operation. These are listed in Columns 1 and 2 of Table S1, linked by their energy codes and descriptions back to the EET and EFM. Some forces were discovered twice, once in the EFM and again in the Internal-External branch of the EET. This is quite acceptable, however they are not listed twice in the table (at least for this reason). As previously noted, what is much less acceptable is missing a stressing force that should be considered. In Column 3 the EET and EFM energies are translated into fixture energies to be employed in the MEOST setup, along with the unit of measure in Column 4. These are really “intermediate” energy descriptions based on various research papers and the industrial standards referenced [25]–[55] in Column 9. Using those same references, Columns 5 and 6 provide max and min design values for each energy. Normally, operating limits would be specified as well. However operating limits are customer dependent and not available at this time. The MEOST experiment developed here would test directly up to the design limits, which include the safety margins beyond the operating limits. Column 7 indicates which design limit extreme, high or low, is unfavorable in terms of aggravation. That extreme is then used in Column 8 to determine the MPOSL, which for our purposes here is simply taken as ∼70% above the design limit, 1/3 of the way to the destruct limit, or 70% of the Max-Min difference, whichever is less. In cases where the worst setting is within the min/max range (“Middle”), that setting is entered in the MPOSL column and no additional stressing is possible.

All the energies found in the EET and EFM are listed in the table. The table objective is to group energies in a practical way for simulation in the MEOST fixture. Energy units and limits for piping, where available, were developed based on research and other specifications or standards.

Several features of the MEOST table are worth pointing out. Some EET and EFM energies require multiple mechanisms to simulate, for example galvanic effects depend on both metal type and sea water velocity. We limit some energies in the table, such as “Linear X-Y soil forces” to non-destructive levels because the MEOST experiment specifically excludes Useful Life energy spikes. Some Column 3 lines with the same fixture energy are unfavorable at both max and min setting, such as oil temperature which affects viscosity and thermal gradients on the high end, but carbon dioxide and sulfate reducing bacteria on the low end. Other lines in the table have no max/min data. Such energies are “leakages” discovered by the systematic EET and EFM methods we have employed. They should be included, or at least considered, in a complete pipe MEOST reliability study. “Residual weld stresses” result from imperfect welds which come from dozens of factors, like dirt, gas, hydrogen, sulfur, slag inclusion, cooling speed, etc. For this reason, weld stresses would require a completely separate study. However the pipe MEOST experiment itself should include pipes welded with permissible quality extremes to look for sensitivity and interactions.

For testing we also need time profiles for each energy. Profiles are not shown in Table S1, however, where different profiles for the same energy type are needed based on the EET and EFM energies, these are shown as separate lines in the table. For example, “Solid (soil, rock impulses)”, “Ocean waves”, “Seawater vortex” and “Pump vibrations” all result in oscillations, but with different frequencies and amplitudes. A computer can generate appropriate time-dependent signals to represent the sum of all these forces. Other seemingly more complex energies such as “chemistries” that create corrosion would cycle as a group between the most and least favorable settings.

Within the Operating Rectangle time profiles should reflect normal behavior with frequent excursions (to accelerate time) up to the operating limits of each variable (design limits in this proposal). In overstressing, rates of change should be increased in the same proportion as the forces themselves are increased. Unlike in manufacturing, time behavior is unlikely to be cyclic. For example some functions are relatively static, e.g., a pipe resting on the seabed, while occasional impulse forces will arise if the seabed shifts. Other forces will vary continuously, like ocean hydraulics, chemistry and temperature. Yet other cases are random in nature, such as mechanical impacts, while still others are relatively large and constant over time, such as internal oil pressure and possibly vibrations.

The MEOST table is organized by grouping similar Column 3 fixture energies in order to simplify the physical design of the MEOST setup. In some cases it is possible to simulate an effect in multiple ways. For example “Seawater current” may be simulated directly with water velocity or by applying an equivalent horizontal force in the X direction. We have chosen to show only one, however the experimentalist may select any other for the sake of convenience. Inspection of the table groupings suggests that perhaps a dozen or so different energy actuators would be sufficient to simulate the 41 EET and EFM energies.

As the final planning step, even the intermediate energy groupings in Column 3 need the MEOST hardware design team to make one more translation into actual physical mechanisms, such as mechanical (e.g., X-Y pistons, straps to apply compression and torque, vibration, bumping, etc.), hydraulic, thermal, chemical, abrasives, electrical, etc. The max/min design and MPOSL limits of each actuator would be updated along with this final conversion. In doing so, it will be seen that under certain circumstances some energies may become insignificant, such as wave height when a pipe is laid a thousand meters below the surface, while others such as external compressive pressure from a “Vacuum spike” following a point of rapid blockage would become enormous. The opposite would be true when the pipe is near the sea surface. This final, most practical, step is not discussed in this paper.

## Conclusions

A systematic technique for discovering energies that create system reliability risks was proposed. The conventional accelerated lifetime testing approach employed in the civil engineering industry was upgraded by introducing MEOST.

Two significant creations were established in developing the testing methodology. First, an innovative Expansion Tree was built following six decomposition principles based on physics, geometry and logic. We call this an Energy Expansion Tree (EET) because it focuses on discovering external (environmental) energies and forces resulting from external-to-internal energy domain differences. The approach assures more complete coverage of energies which act to degrade a system over time.

Second, the conventional function flow model is augmented to clearly reveal internal energy storage, transfer, leakage and other forces associated with the primary system functions. These “side effect” energies combine with the external energies to complete the list of energy sources which may contribute to reliability failures individually or through interactions.

As an example, the new methods were qualitatively applied to the piping subsystem of a civil engineering Subsea Pipeline Project. Completing the analysis and finalizing the MEOST experiment for this application would require suitable industry experts. However, the methodology is clearly applicable to a much wider field than subsea pipelines.

## Supporting Information

Table S1 **MEOST table, experimental layout with grouped energies.**
(TIF)
