# Supplementary figures and images for: Methodology to Improve Design of Accelerated Life Tests in Civil Engineering Projects
Source: PLoS One. 2014 Aug 11;9(8):e103937. doi: 10.1371/journal.pone.0103937 (PMC4128810; doi:10.1371/journal.pone.0103937)

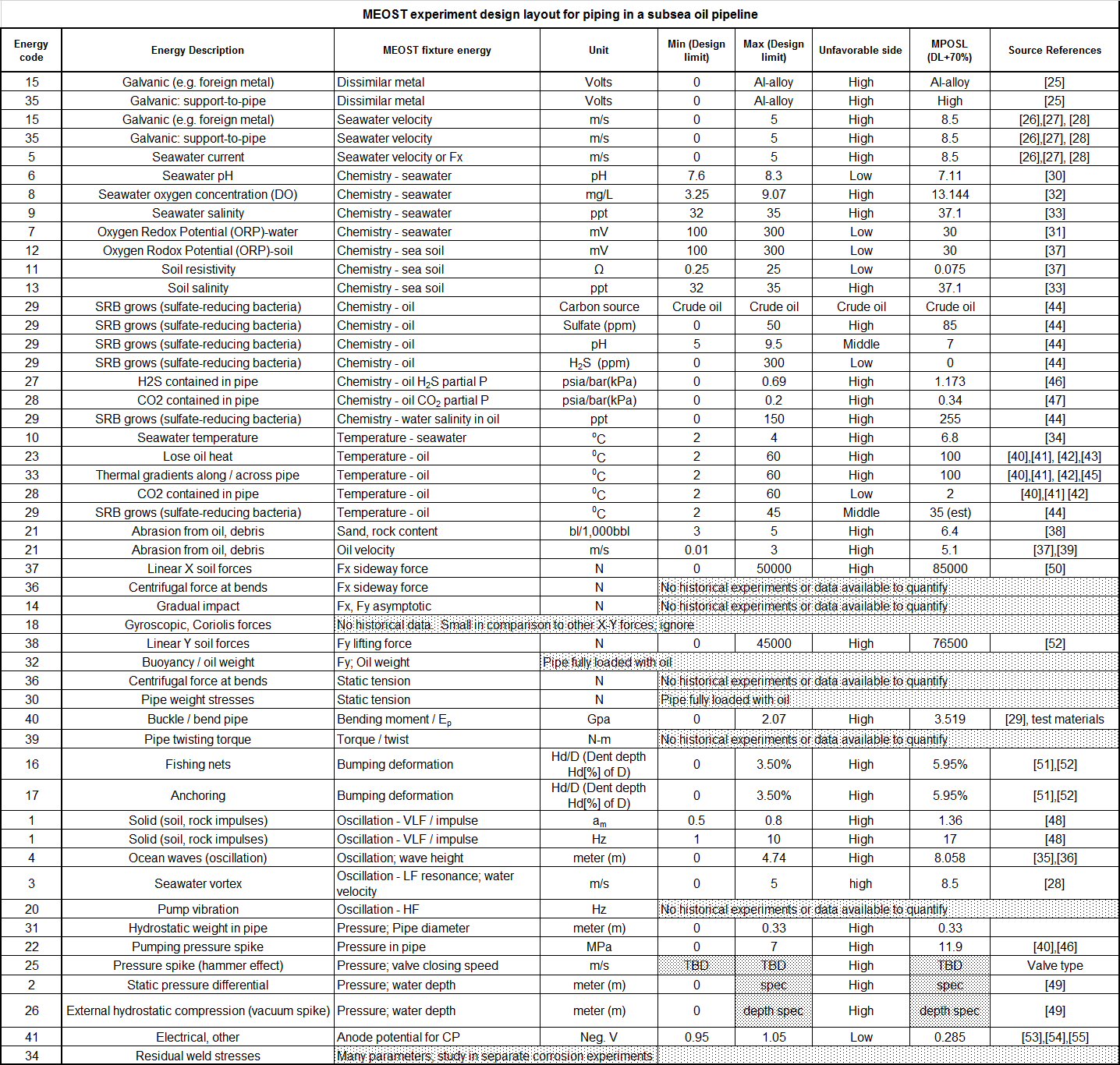

Supplement: Table S1 — MEOST table, experimental layout with grouped energies. (TIF) [file pone.0103937.s001.tif]
